# Identification of a familial complex chromosomal rearrangement by optical genome mapping

**DOI:** 10.1186/s13039-022-00619-9

**Published:** 2022-09-21

**Authors:** Yang Yang, Wang Hao

**Affiliations:** 1 Prenatal Diagnosis Center, Hangzhou Maternity and Child Care Hospital, #369 Kunpeng Road, Shangcheng District, Hangzhou, 310008 Zhejiang China; 2 Department of Cell Biology and Medical Genetics, School of Medicine, Zhejiang University, Hangzhou, Zhejiang China

**Keywords:** Complex chromosomal rearrangement, Fluorescence in situ hybridization, Chromosomal microarray analysis, Prenatal diagnosis, Optical genome mapping

## Abstract

**Background:**

Complex chromosomal rearrangements (CCRs) are rare chromosomal structural variations, containing a variety of rearrangements such as translocation, inversion and/or insertion. With the development of cytogenetic and molecular genetic techniques, some chromosomal rearrangements that were initially considered to be simple reciprocal translocations in the past might eventually involve more complex chromosomal rearrangements.

**Case presentation:**

In this case, a pregnant woman, who had a spontaneous abortion last year, had abnormal prenatal test results again in the second pregnancy. Applying a combination of genetic methods including karyotype analysis, chromosomal microarray analysis, fluorescence in situ hybridization and optical genome mapping confirmed that the pregnant woman was a carrier of a CCR involving three chromosomes and four breakpoints, and the CCR was paternal-origin. Her first and second pregnancy abnormalities were caused by chromosomal microdeletions and microduplications due to the malsegregations of the derivative chromosomes.

**Conclusions:**

We presented a rare familial CCR involving three chromosomes and four breakpoints. This study provided precise and detailed information for the subsequent reproductive decision-making and genetic counselling of the patient.

## Background

Complex chromosomal rearrangement (CCR) is a rare chromosomal structural abnormality involving three or more breakpoints on at least two chromosomes [1, 2]. Madan [3] classifies CCRs into four categories: (i) type I (the number of breakpoints/the number of involved chromosomes = 1) is usually caused by three-way or four-way translocation; (ii) type II (the number of breakpoints/the number of involved chromosomes > 1) has an inversion; (iii) type III (the number of breakpoints/the number of involved chromosomes > 1) has at least one insertion; (iv) type IV (the number of breakpoints/the number of involved chromosomes > 1): there is one or more derivative chromosomes containing segments from at least three chromosomes.

About 70% CCR carriers are phenotypically normal, but they have a high risk of recurrent miscarriage, subfertility or infertility, and pregnancy abnormalities due to conceiving offspring with unbalanced CCRs [1, 4, 5]. With the development of cytogenetic and molecular techniques, more complex and cryptic chromosomal imbalances have been revealed [6–9]. Optical genome mapping (OGM) has been proven to show efficacy in detecting complex chromosomal structural aberrations [10, 11]. Here, we present a familial CCR identified by OGM.

## Case presentation

**Fig. 1:**
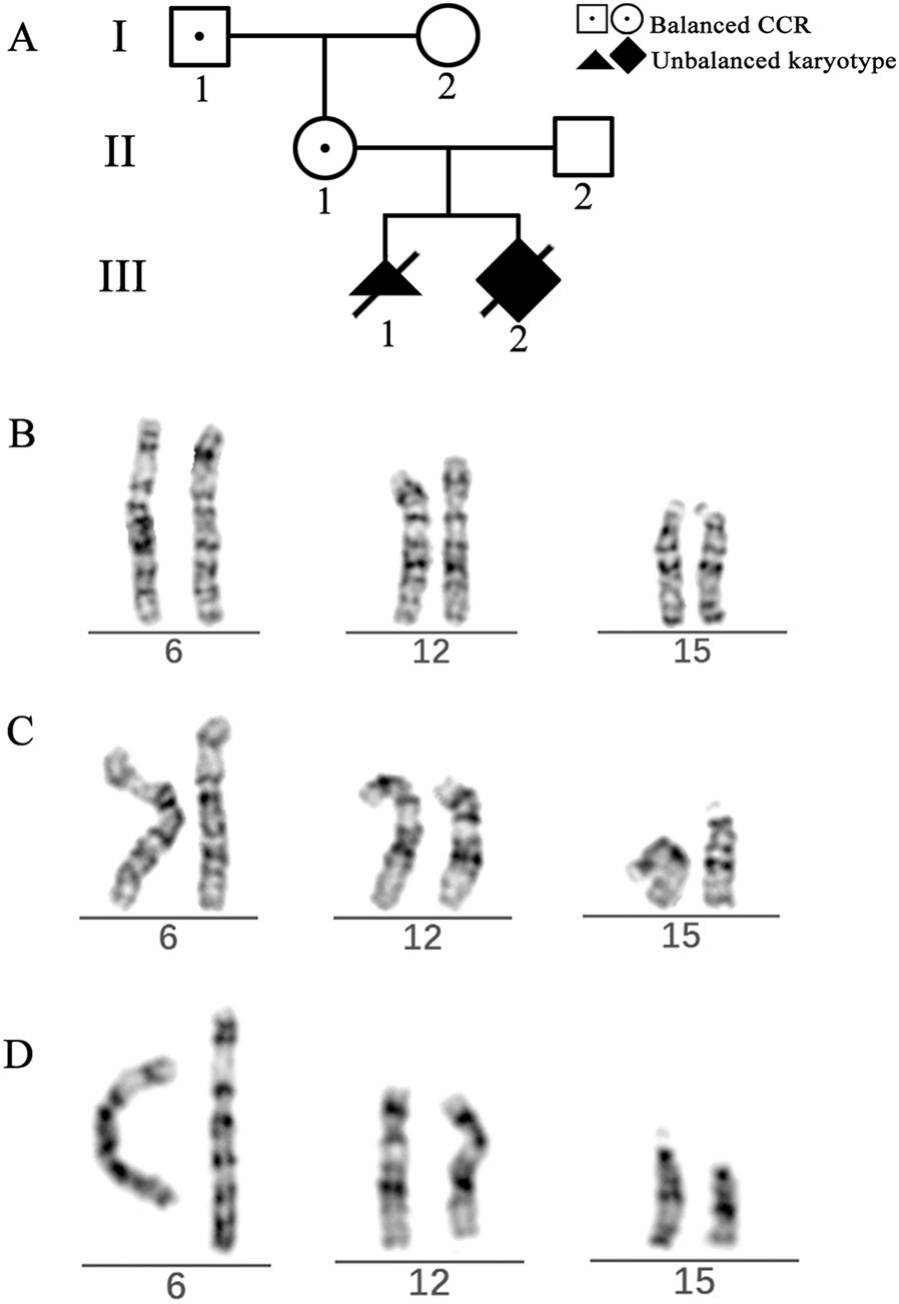
Pedigree and partial karyotypes of the family. **A** Pedigree of the family. **B** Chromosome 6, 12, 15 of III-2. **C** Chromosome 6, 12, 15 of II-1. **D** Chromosome 6, 12, 15 of I-1

A 27 years old woman (II-1) was referred to our center due to the abnormal prenatal screening test results (Fig. 1A). The unconjugated estriol (uE3) level of the maternal serum was low (3.18 nmol/L, 0.69 MoM). The non-invasive prenatal test result showed 9 Mb duplication of 15q26.1q26.3. The patient had a history of spontaneous abortion (III-1) last year, and the CNV-sequencing result of the tissue of the aborted fetus was: seq[hg19] dup(6)(q27) chr6:g.166080000_170920000dup; seq[hg19] del(15)(q26.1q26.3) chr15:g.92820000_102400000del.

Because of the abnormal prenatal test results, the patient underwent amniocentesis. The amniotic fluid sample of the fetus (III-2) was then subjected to karyotype analysis and chromosomal microarray analysis (CMA). Suspected rearrangements were observed in the distal ends of chromosome 6 and 12 (Fig. 1B), but the materials of origin were unknown. The CMA result showed: arr[GRCh37] 12q24.33(131833209_133777562) × 1,15q26.1q26.3(92791507_102429040) × 3. The peripheral blood samples of the parents (II-1, II-2) were obtained to investigate the origin of the structural abnormality. The father (II-2) of the fetus showed a normal karyotype, and structural abnormalities were observed in the mother (II-1). Suspected rearrangements were found in the distal ends of chromosome 6, 12 and 15 (Fig. 1C).

**Fig. 2:**
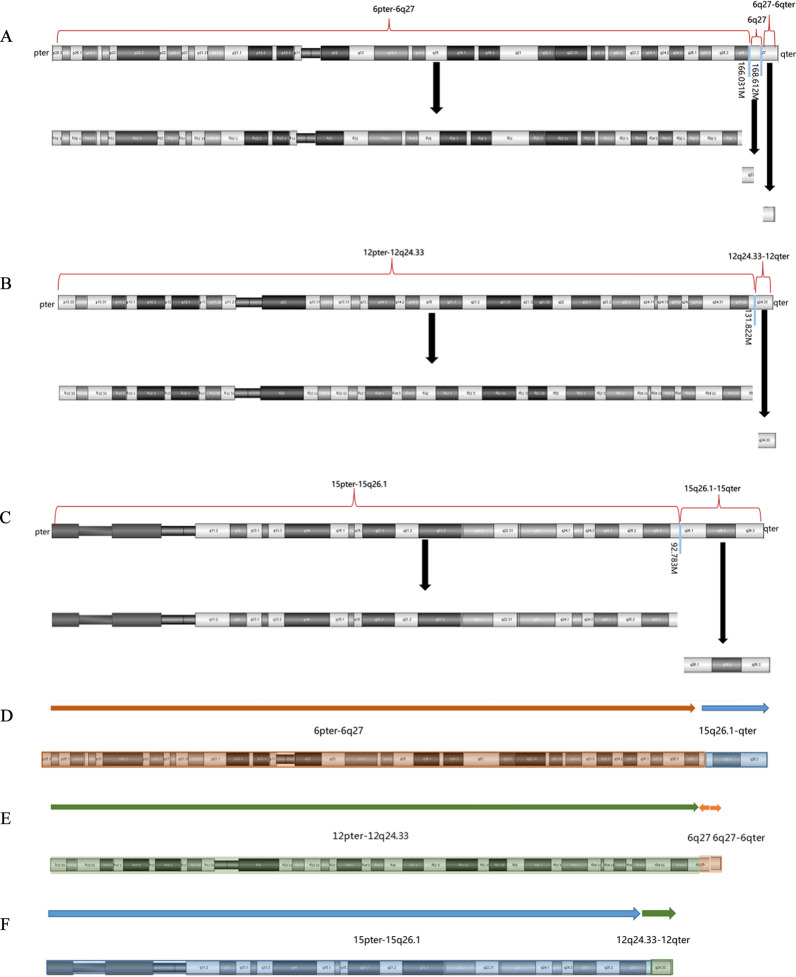
Schematic diagram of the breakages and fusions of chromosome 6, 12 and 15. **A** Chromosome 6 breaks into three segments: 6pter-6q27 (0M–166.031M), 6q27 (166.031M–168.612 M), 6q27-6qter (168.612M–171.016M). **B** Chromosome 12 breaks into two segments: 12pter-12q24.33 (0M–131.822M), 12q24.33-12qter (131.822M–133.840M). **C** Chromosome 15 breaks into two segments: 15pter-15q26.1 (0M–92.793M), 15q26.1-15qter (92.793M–102.516M). **D** The derivative chromosome 6 has resulted from a translocation of the chromosome 15 segment (15q26.1-15qter) to the long arm of chromosome 6 at band 6q27. **E** The derivative chromosome 12 has resulted from a reverse insertion of the segment 6q27 onto 12q24.33, and a translocation of segment 6q27-6qter onto chromosome 12 at 6q27. The arrows indicate the directions of the segments. **F** The derivative chromosome 15 has resulted from a translocation of the segment of chromosome 12 (12q24.33-12qter) to chromosome 15 at 15q26.1

**Fig. 3:**
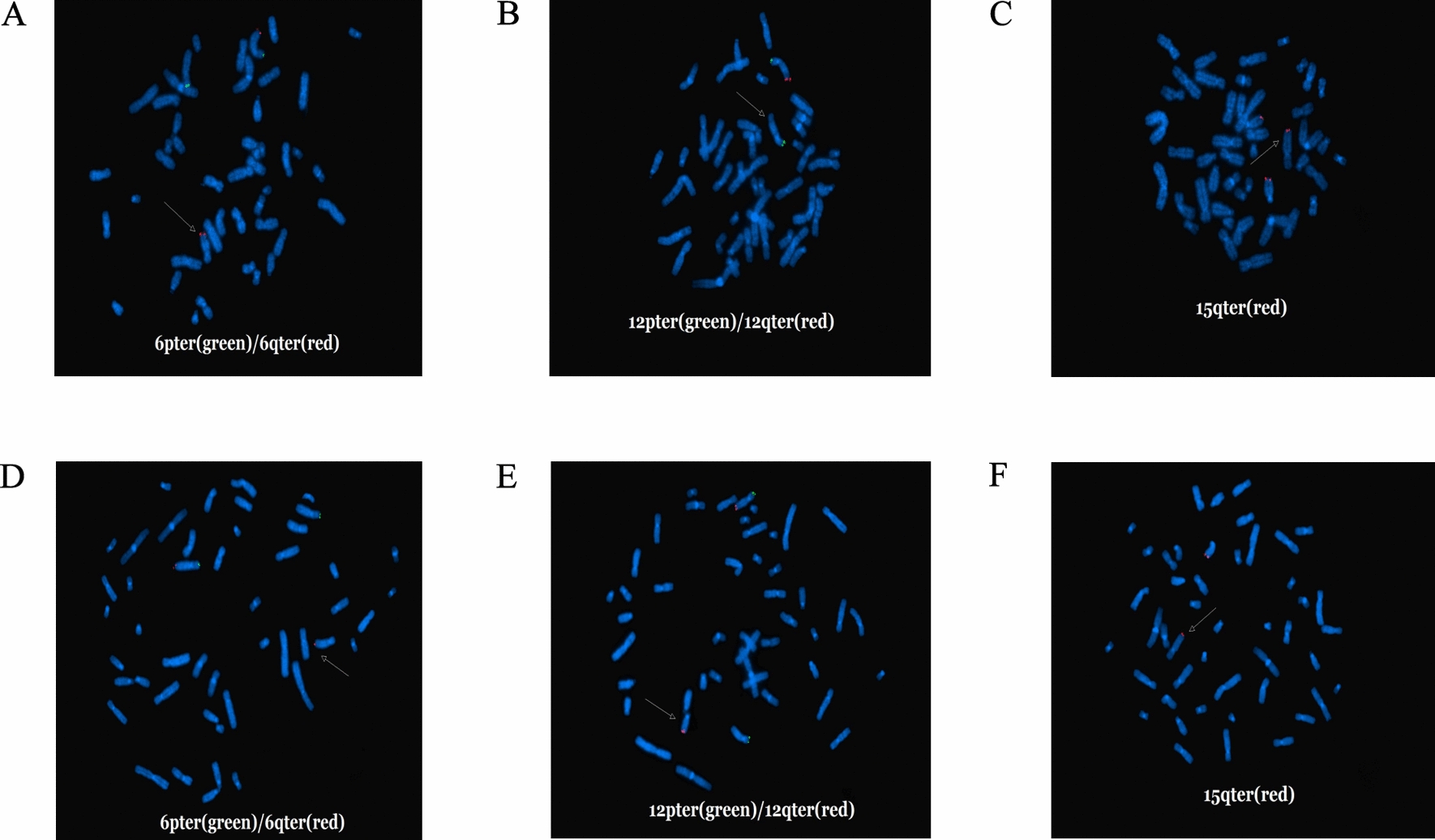
Fluorescence in situ hybridization (FISH) results of III-2 (**A**–**C**) and II-1 (**D**–**F**). A. One 6qter (red) signal was found on the distal end of chromosome 12. **B** One 12qter (red) signal was missing. **C** Three 15qter (red) signals was observed, and one signal was found on the distal end of chromosome 6. **D** One 6qter (red) signal was observed on the distal end of chromosome 12. **E** One 12qter (red) signal was found on the distal end of chromosome 15. **F** One 15qter (red) signal was observed on the distal end of chromosome 6

The peripheral blood of the mother (II-1) and the cord blood of the fetus (III-2) were subjected to OGM. The mother (II-1) had three derivative chromosomes (chromosome 6, 12 and 15), and the fetus (III-2) had two derivative chromosomes (chromosome 6 and 12) inherited from the mother. The breakages and fusions of the chromosomes were identified by OGM (Fig. 2). Fluorescence in situ hybridization (FISH) analysis verified the results (Fig. 3). The fetus (III-2) had the same derivative chromosome 6 and 12 with the pregnant woman (II-1) and two copies of normal chromosome 15. Therefore, the pregnant woman (II-1) was a carrier of the balanced CCR, and the fetus (III-2) had the unbalanced CCR. Because the reverse insertion of the segment 6q27 onto 12q24.33 was submicroscopic (2.581M) and 6q27 was not subdivided into sub-bands, this reverse insertion could not be described by karyotype. In brief, the karyotype of II-1 was 46,XX,der(6)t(6;15)(q27;q26.1)dpat,der(12)t(6;12)(q27;q24.33)dpat,der(15)t(12;15)(q24.33;q26.1)dpat, and the karyotype of III-2 was 46,XX,der(6)t(6;15)(q27;q26.1)dmat,der(12)t(6;12)(q27;q24.33)dmat.

The peripheral blood samples of the parents (I-1, I-2) of the pregnant woman (II-1) were obtained and underwent karyotyping. The father (I-1) of the pregnant woman had the same karyotype as his daughter (Fig. 1D), and the mother (I-2) of the pregnant woman had a normal karyotype.

## Discussion

**Fig. 4:**
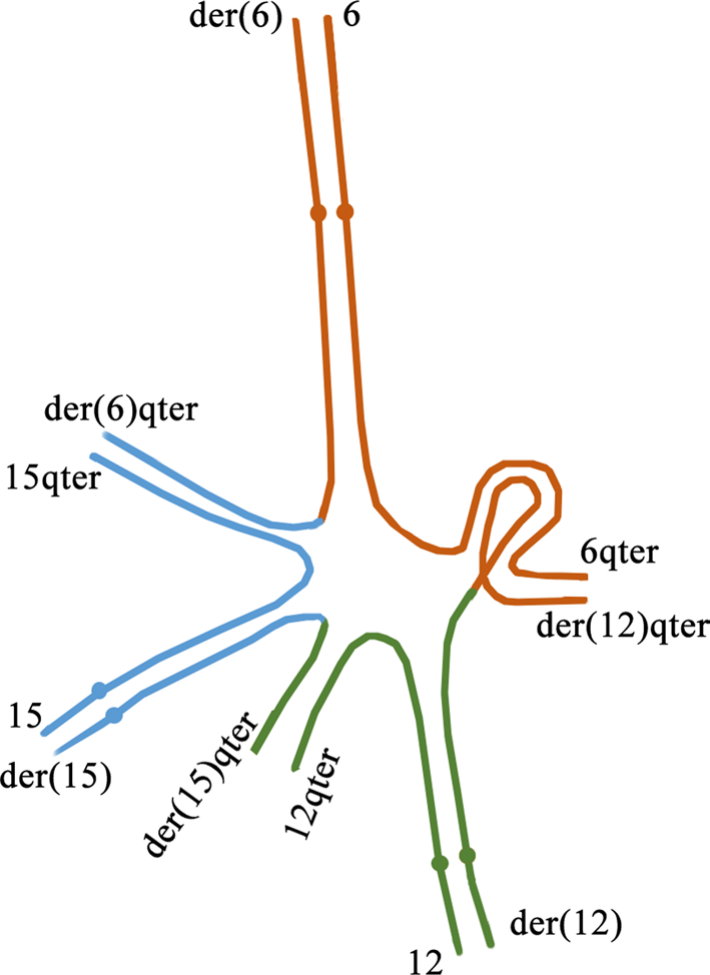
Schematic diagram of the hexavalent structure formed by chromosome 6, 12, 15 and derivative chromosome 6, 12, 15

Most CCR cases are de novo in origin [1]. The majority of familial CCRs are transmitted through females, and a very few male transmission CCR cases have been reported [1, 2]. This is because CCRs would impair the spermatogenesis or lead to meiotic arrest [12–14]. In the present case, the CCR was transmitted through both male (I-1) and female (II-1). During meiosis I, the derivative chromosomes would form a hexavalent structure (Fig. 4). This structure allows the pairing of the chromosomes, where only small segments around the breakpoints are not fully paired. In this case, the theoretical modes of segregations (3:3, 4:2, 5:1, 6:0) would produce many different gametes [2]. However, the most frequent mode is symmetric (3:3) segregation, resulting in theoretically 20 kinds of gametes including one normal, one balanced and 18 unbalanced gametes [2, 3]. Therefore, we could conclude from the CNV-sequencing result of III-1 that III-1 had the derivative chromosome 12, 15 and two copies of normal chromosome 6, leading to the unbalanced chromosomal rearrangement.

Because III-1 had three copies of 6q27 and III-2 had three copies of 15q26.1q26.3, uniparental disomy (UPD) existed in III-1 and III-2. Since UPD(6)pat would lead to transient neonatal diabetes mellitus and 6q27 is not critical for the disorder development [15], III-1 with segUPD(6)mat in 6q27 didn’t have the risk of the imprinting-caused disorder. UPD(15)mat is associated with Prader Willi syndrome (PWS), but segUPD(15)mat in 15q26.1 to 15q26.3 is not critical for PWS development [15]. Therefore, III-2 might not be affected by imprinting.

The recurrence risk of CCR is difficult to estimate, because each CCR is unique and needs to be studied separately [5, 16]. In general, the risk is related to the nature of the CCR, the number of involved chromosomes and breakpoints [2]. It is known that the severity of abnormal pregnancy outcome grows with the increasing number of involved chromosomes and breakpoints [17]. In the present study, the parents decided to terminate the pregnancy, and we suggested preimplantation genetic diagnosis of embryos for their future reproductive decisions.

In this study, we applied multiple techniques to reveal the complicated breakages and fusions of the chromosomes. Karyotyping could not identify submicroscopic rearrangements (< 5 Mb), while CMA could not detect balanced translocations [4, 5]. FISH analysis needs specific probes and complex procedures. OGM is a long DNA molecule-based technique which could recognize whole-genome-wide structural variations [18]. It is an optimal method for detecting chromosomal structural variations, especially for the analysis of CCRs [19–21]. In the present case, OGM identified a more complicated rearrangement than initially appreciated, and the result was validated by FISH. These methods applied in the study are supplementary to each other, and identified a rare CCR event in this family, which greatly assisted the prenatal diagnosis and genetic counselling. Combining multiple molecular and cytogenetic techniques would help reveal cryptic structural aberrations such as small segment translocations or inversions and help understand the underlying genetic etiology of recurrent miscarriages or pregnancy abnormalities.

## Data Availability

The datasets used and/or analyzed during the current study are available from the corresponding author on reasonable request.
